# An easy method for the clear detection of beige fat UCP1 by Western blotting

**DOI:** 10.1080/21623945.2019.1693746

**Published:** 2019-11-22

**Authors:** Min-Jung Park, Chang-Min Lee, Dong-il Kim

**Affiliations:** a Department of Veterinary Physiology, College of Veterinary Medicine, Chonnam National University, Gwangju, Korea; b Department of Veterinary Laboratory Medicine, College of Veterinary Medicine, Chonnam National University, Gwangju, Korea

**Keywords:** Acetone precipitation, beige adipocytes, white adipose tissue, UCP1, cleaning method

## Abstract

Beige adipocytes, which consume energy mainly in an uncoupling protein 1 (UCP1)-dependent manner, are risen in white adipose tissue (WAT) depots. Since beige adipocyte development is gaining attention as a potential strategy for conquering obesity, worldwide researchers are making efforts to study its biological aspects. However, assessing UCP1 protein levels in beige adipocytes is challenging because of the high level of lipid contaminants in WAT. This study showed that an acetone precipitation method had advantages over conventional methods for eliminating lipid contaminants, achieving clear Western blot bands for WAT proteins, especially UCP1. Our results suggest that the acetone precipitation cleaning method could be useful for the clear analysis and precise evaluation of WAT proteins.

## Introduction

The presence of thermogenic adipocytes in adult humans was identified by three independent studies in 2009 [1–3]. The cells observed in adults in those studies were considered to be similar to murine brown adipocytes, however, later studies revealed them to be a distinct type of thermogenic adipocytes, called beige adipocytes, which were mainly observed previously in murine subcutaneous (SQ) white fat depots following thermogenic stimulation [4–6]. Current studies have focused on uncovering mechanisms which can activate beige adipocytes because activation is anticipated to be a potential therapeutic approach to combating obesity.

Thermogenic stimulations, such as cold conditions and/or β3-adrenergic receptor activation, give rise to the formation of beige adipocytes in SQ fat depots[4]. Similar to brown adipocytes, beige adipocytes express uncoupling protein 1 (UCP1), a mitochondrial inner membrane protein which generates heat by dissipating the proton gradient without producing ATP [7]. Therefore, measuring the UCP1 levels in SQ fat depots has been widely used to predict the thermogenic activity of beige adipocytes. In the murine model, inguinal fat depots are the biggest white SQ depot, easy to dissect, and the place where the stimulation-induced differentiation of beige adipocytes is most abundant [4,8]. One of the easiest ways to estimate UCP1 levels in inguinal depots is by measuring *Ucp1* mRNA levels by quantitative PCR. However, a previous study reported that UCP1-mediated thermogenic activity was regulated by post-transcriptional stabilization[9]. Moreover, appropriate splicing of *Ucp1* mRNA was required for the translation to functionally-correct UCP1, suggesting the importance of the direct measurement of UCP1 protein levels of the expected size rather [10].

For the measurement of UCP1 protein expression, Western blotting (WB) using a UCP1-specific antibody is usually performed. However, WB of white fat depots, such as inguinal fat, has considerable limitations because the tissue lysates of white fat contain numerous lipid components that disrupt the clear interpretation of the WB results. To address the issue of lipid contamination, we adopted the well-established acetone precipitation method to remove residual lipids [11,12]. Here, we found that the cleaning method using acetone, which is reasonable and convenient, was enough to detect clean UCP1 protein bands of beige adipocytes without any disturbance of lipid contamination and following undesirable protein dragging.

## Materials and methods

### Animal experiments

Mouse experiments were carried out in accordance with the protocol approved by the Institution Animal Care and Use Committee of Chonnam National University (approval No. CNU IACUC-YB-R-2019-10). All mice were maintained at a 12 h light/dark cycle with *ad libitum* food and water. For the cold climate studies, C57BL/6J mice (Jackson Laboratory, #000664; Bar Harbour, ME, USA) were single-housed at 4 °C for 4 days or at room temperature (24°C) as a control group. For the high-fat diet (HFD) studies, eight-week-old mice were fed either a normal chow diet (control group) or an HFD (60% of calorie from fat; Research Diets) for 12 weeks. At the end of the experimental period, the mice were euthanized by CO_2_ inhalation. Interscapular brown adipose tissues (iBATs) from UCP1 knock-out (UCP1 -/-; Jackson Laboratory, # 003124; Bar Harbour, ME, USA) and wild-type mice (UCP1+/+) were used as negative and positive controls, respectively, in UCP1 Western blots.

### Protein extraction with or without cleaning method

Tissues were homogenized in RIPA buffer [140 mM NaCl, 10 mM Tris pH 8.0, 0.1% sodium deoxycholate, 1 mM EDTA, 1% Triton X-100, 0.1% sodium dodecyl sulphate (SDS), and 1 mM phenylmethylsulphonyl fluoride] or adipocyte lysis buffer (500 mM NaCl, 50 mM Tris pH 7.4, 1% NP40, 20% glycerol, 5 mM EDTA, and 1 mM phenylmethylsulphonyl fluoride) containing protease inhibitor cocktail (Sigma-Aldrich, 11836153001; St. Louis, MO, USA). Protein concentrations were measured using a DC Protein Assay kit (Bio-Rad, 500–0112) according to the manufacturer’s instructions. For conventional method samples, 30 μg of protein (for one well) was mixed with 5x SDS sample buffer [60 mM Tris-HCl (pH6.8), 25% glycerol, 0.1% bromophenol blue, 2% SDS, and 2% β-mercaptoethanol] and boiled for 3 min. For the cleaning method samples, four volumes of 100% cold acetone were added to 60 μg of proteins (for one well), mixed by inverting, and incubated at −20°C for at least 30 min or overnight. After centrifugation for 10 min at 15,000 × g, the supernatants were discarded and the pellets were washed with 80% cold acetone. The sample was centrifuged again for 5 min at 15,000 × g, the supernatants were discarded, and the residual solution was evaporated at room temperature for 10 to 20 min. After drying, 2x SDS sample buffer was added to the pellet, mixed by vortexing, and boiled for 3 min.

### Western blotting

The denatured protein lysates were loaded onto SDS-PAGE gels and transferred to nitrocellulose (NC) membranes. Antibodies to UCP1 (Abcam, ab10983), Actin (Cell Signalling Technology, 8457), HSP90 (Cell Signalling Technology, 4874), PPARγ (Perseus Proteomics, PP-A3409A), and Perilipin 1 (Vala Sciences, 4854) were diluted according to manufacturers’ recommendations and the NC membranes were incubated with each antibody at 4 °C overnight. After washing with TBS-T (20 mM Tris, 150 mM NaCl and 0.1% Tween20), the membranes were incubated with secondary anti-rabbit (Cell Signalling Technology, 7074) or anti-mouse (Cell Signalling Technology, 7076) IgG antibodies for 2 h at room temperature. Actin and HSP90 were used as loading controls. The immunoreactive bands were detected with ECL Lumi Pico (Dogen, DG-WPAL250) using ImageQuant LAS 4000 (GE Healthcare Life Sciences).

## Results

### Improved resolution of inguinal WAT lysates extracted in RIPA buffer using the cleaning method

**Figure 1.**
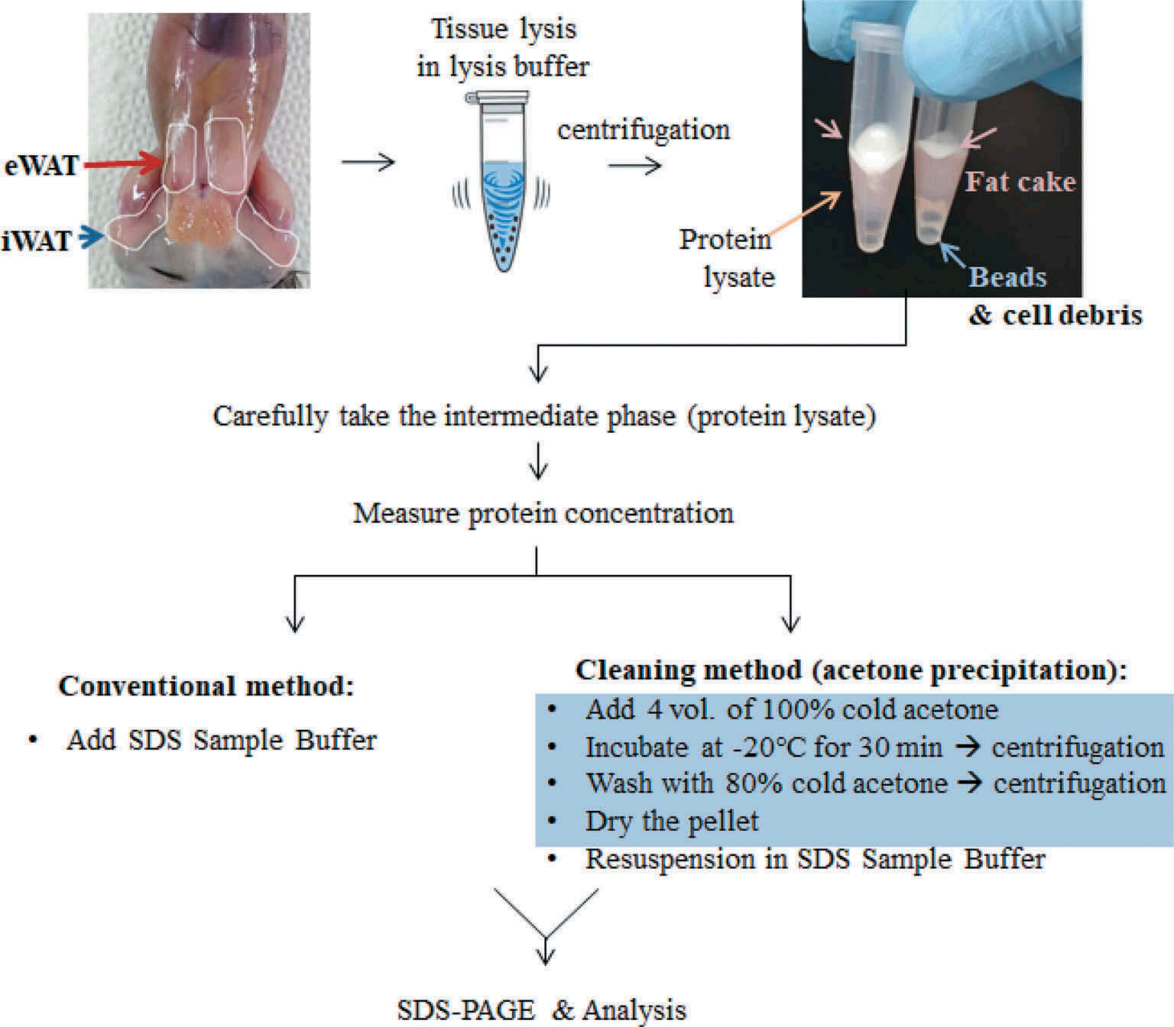
Schematic diagram of protein sample preparation from fat tissues for Western blotting. Crude proteins from lipid-rich tissues, such as epididymal or inguinal white adipose tissues (eWAT or iWAT, respectively), were prepared by chemical (lysis buffer) and physical (homogenization by shaking beads) lysis followed by centrifugation. After centrifugation, the lipids (fat cake) are overlaid on top of the protein-containing supernatant. To eliminate lipids from the protein fraction, an acetone precipitation method was adopted, as indicated in the blue box (see the details in the methods section).

**Figure 2.**
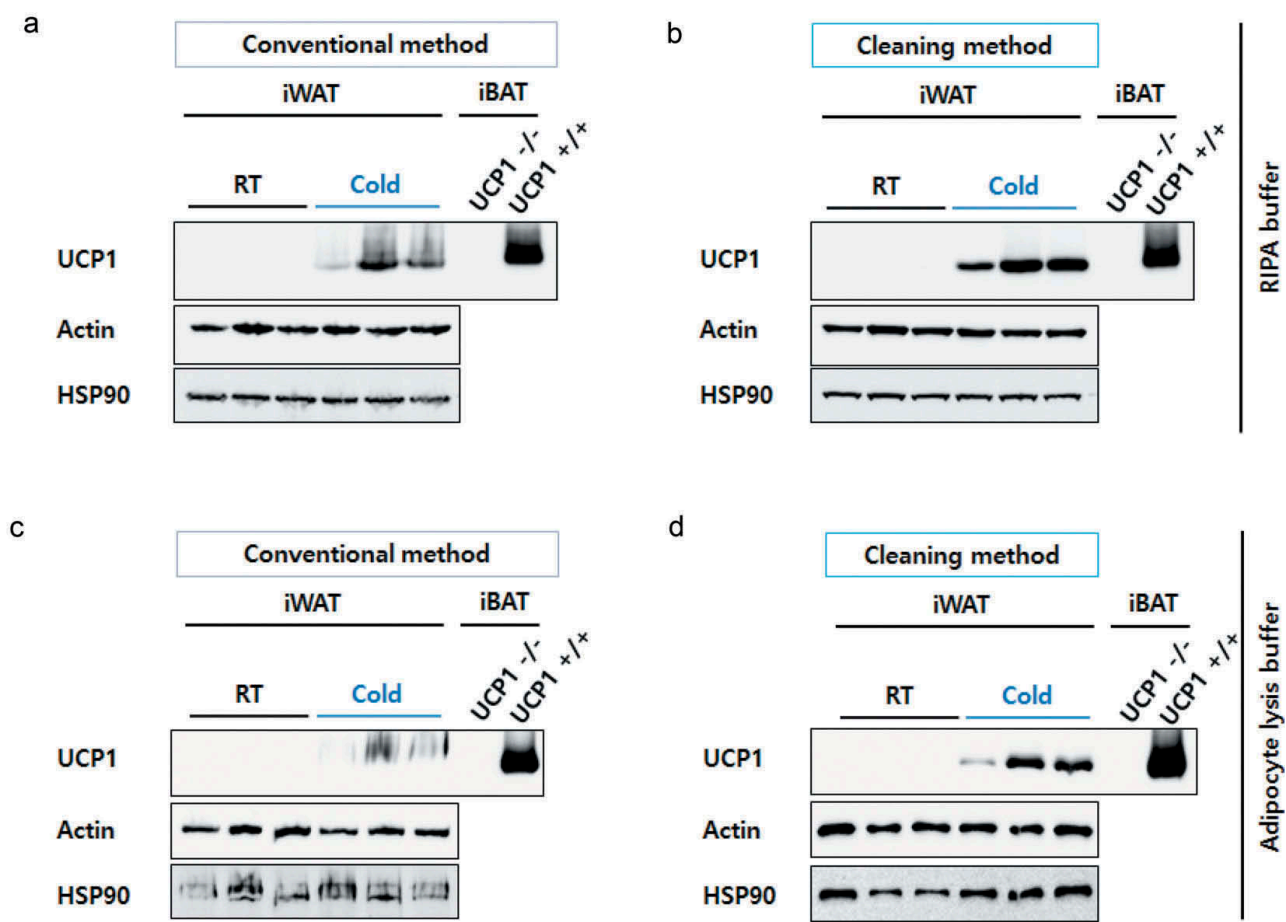
Resolution comparison for iWAT proteins. iWAT from mice exposed to cold conditions (Cold, *n*= 3) or housed at room temperature (RT, *n*= 3) were lysed in RIPA buffer (a) or adipocyte lysis buffer (b) and prepared by the conventional method (30 μg/well) or the cleaning method (60 μg of starting weight/well). Blots were probed with antibodies against UCP1 or two different loading controls, Actin and HSP90. Protein lysates from interscapular brown adipose tissue (iBAT) from UCP1 knock-out (UCP1 -/-) or control mice (UCP1 +/+) were prepared by a conventional method and used as a negative or positive control for UCP1, respectively. Compared to iWAT, only 10% of the iBAT lysate was loaded (3 μg/well).

To develop beige adipocytes, C57BL/6J mice were housed in a cold climate for 4 days. Control mice were housed at room temperature. Inguinal fat depots (inguinal white adipose tissue, iWAT) were dissected and protein lysates were prepared in RIPA buffer according to the conventional or the cleaning method (Figure 1). During UCP1 detection, iBATs from UCP1 knock-out (UCP1 -/-) and wild-type (UCP1 +/+) mice were used as negative and positive controls, respectively. The samples were analysed by WB with UCP1 antibody (ab10983). As expected, the UCP1 levels were highly increased in the iWAT of mice housed in cold conditions (Figure 2(a)). However, the UCP1 bands of iWAT were smeary and unclear because of the unavoidable presence of lipids, even after conventional protein preparation. Compared to these, the UCP1 signals from the iWAT lysates prepared using the cleaning method were markedly clearer (Figure 2(b)). Actin and HSP90 immunoblots that were used as loading controls also became sharp (Figure 2(a,b)).

### Improved resolution of inguinal WAT lysates extracted in adipocyte lysis buffer using the cleaning method

To further confirm the effectiveness of the cleaning method, we lysed iWAT in adipocyte lysis buffer, which is also frequently used for fat tissue lysis [13], although RIPA buffer is most widely used to extract proteins from diverse tissues. Similar to the results observed with RIPA buffer, clearer bands for UCP1 were detected in samples prepared by the cleaning method (Figure 2(c,d)), suggesting the cleaning step improved the resolution of Western blot proteins from fat-rich tissues.

### Improved resolution of epididymal WAT lysates extracted in RIPA buffer using the cleaning method

**Figure 3.**
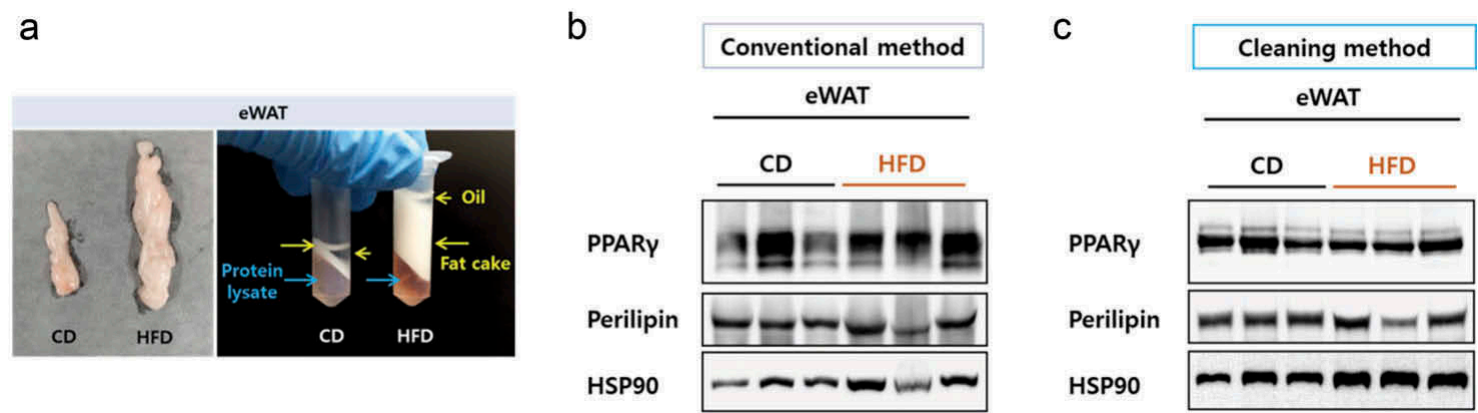
Resolution comparison for eWAT proteins. (a) Left, size comparison of one side of eWAT from mice fed a chow diet or a high-fat diet. Right, separated layers after a centrifugation during a protein preparation. Blue arrows indicate protein lysate component and yellow arrows indicate fatty components. (b) eWAT lysed in RIPA buffer and protein lysates were prepared by either the conventional (b) or the cleaning method (c). Blots were probed with antibodies against PPAR gamma or perilipin and HSP90 as a loading control.

Obese mice possess a strikingly high level of fatty component, especially in epididymal WAT (eWAT). To examine if the cleaning method could be applied to improve the case, we tested eWAT from mice fed either a chow diet or a high-fat diet (HFD) for 12 weeks. The volume of the eWAT from the HFD mice became remarkably high and during protein preparation, the fat cake layer plus the overlying oil layer were very deep following centrifugation, making separation of the protein fraction difficult (Figure 3(a)). Despite the careful separation, protein samples prepared by the conventional method were not clean enough and the immunoblot bands for PPARγ and Perilipin were smeared. On the other hand, the immunoblot bands became much clearer after the cleaning step (Figure 3(b,c)), suggesting that the cleaning method can also be effective for detecting eWAT proteins.

## Discussion

Discovering the existence of thermogenic adipocytes in adult humans opened a new era of hope for overcoming obesity [14]. It is noteworthy that thermogenic adipocytes in adults are similar to beige adipocytes, rather than brown adipocytes, in murine [6]. Thus, studies on the mechanism of beige adipocyte generation, also known as browning, could be related to human health. Unlike classical brown adipocytes clustered in specific depots (e.g. iBAT), beige adipocytes are induced by stimulation and spread in SQ depots in the entire body. Interestingly, beige adipocytes arise from distinct precursor cells and these cells are considered to be differentiated most abundantly in iWAT [15].

Compared to the other tissues, WAT contains a huge amount of lipids that potentially contaminate the protein fraction. When the mice are housed in the cold, sympathetic nerve-mediated adrenergic receptor activation (especially β3) induces, not only browning but also lipolysis, converting triglycerides to fatty acids [16]. These fatty acids are required for UCP1 activation [17]. Despite highly active lipolysis, iWAT lysates still contained a lot of lipids and were relatively opaque, even after centrifugation (Figure 1; see the fat cake in the right tube). As shown in Figure 2, lipid contamination of the lysates may not significantly impact the interpretation of the results, when we compare UCP1 bands drastically changed such as in mice housed at room temperature versus cold conditions. However, when mild changes in UCP1 are present, such as in wild-type versus some protein knock-out mice, it is not surprising that immunoreactive bands smearing and dragging make the results inconclusive. Thus, elimination of the lipid components from iWAT protein lysates is a critical step for better evaluation of browning.

The cleaning method is not limited to improving the detection of the UCP1 protein. Current studies found that UCP1-negative or -independent beige adipocytes also contributed to thermogenesis through a PR domain containing 16 (PRDM16)-dependent manner [18–20]. Therefore, the cleaning method used in the present study could be used to detect PRDM16 in iWAT. In addition to use with iWAT, this method could also be effectively adopted for detecting proteins in eWAT, which has a pivotal role in fat storage and contains more lipids, especially when obese (Figure 3). Moreover, another important advantage of using the cleaning method is that users are able to determine the final sample volume, which is beneficial for the detection of weak signals.

Different labs and individual senior scientists may have their own specific methods to minimize the deleterious effects of fatty components in protein lysates. Twice centrifugation of lysate, for instance, could reduce contamination. Using a narrow pipette tip to obtain the protein fraction under the lipid layer requires manual dexterity and may not be a reproducible solution for removing residual and unavoidable lipids, whereas the acetone cleaning method can help even beginners achieve clean WB bands. Here, we demonstrated that a cleaning method using acetone precipitation was used effectively to eliminate lipid contaminants in WAT protein lysates.
